# Multidisciplinary Delphi consensus on malignancy screening in patients with common variable immunodeficiency

**DOI:** 10.3389/fimmu.2026.1897051

**Published:** 2026-08-17

**Authors:** Marta Dafne Cabanero-Navalon, Ana de Andrés-Martín, Arnau Antolí Gil, Carmen Bracke, Juan Luis Carrillo-Linares, ML Cos, Mercedes Gasior, Jorge Gil-Niño, Luis Ignacio González-Granado, Nuria López Osle, Ángel Robles-Marhuenda, Rosario Sánchez Martínez, Silvia Sánchez-Ramón, Pere Soler-Palacin, Xavier Solanich, Miguel Ángel Torralba-Cabeza, José Ramón Aparicio Tormo, Laia Alsina, Pedro Moral Moral

**Affiliations:** 1 Primary Immunodeficiency Unit, Department of Internal Medicine, La Fe University and Polytechnic Hospital, Valencia, Spain; 2 Department of Immunology, Ramón y Cajal University Hospital, Madrid, Spain; 3 Department of Internal Medicine, Bellvitge University Hospital, L’Hospitalet de Llobregat, Barcelona, Spain; 4 Adult Primary Immunodeficiency Unit, Bellvitge University Hospital, L’Hospitalet de Llobregat, Barcelona, Spain; 5 Bellvitge Biomedical Research Institute (IDIBELL), L’Hospitalet de Llobregat, Barcelona, Spain; 6 Fundació Lluita contra les Infeccions, Germans Trias i Pujol University Hospital, Badalona, Spain; 7 Department of Infectious Diseases, Germans Trias i Pujol University Hospital, Badalona, Spain; 8 Department of Internal Medicine, Virgen de la Victoria University Hospital, Málaga, Spain; 9 Instituto de Investigación Biomédica de Málaga y Plataforma en Nanomedicina, IBIMA Plataforma BIONAND, Málaga, Spain; 10 Primary Immunodeficiencies Functional Unit, Hospital del Mar, Barcelona, Spain; 11 Department of Internal Medicine, Hospital del Mar, Barcelona, Spain; 12 Department of Hematology, La Paz University Hospital, Madrid, Spain; 13 Department of Internal Medicine, 12 de Octubre University Hospital, Madrid, Spain; 14 Department of Pediatrics, Primary Immunodeficiencies Unit, 12 de Octubre University Hospital, Institute for Health Research (imas12), Madrid, Spain; 15 Complutense University School of Medicine, Madrid, Spain; 16 Internal Medicine, Biobizkaia Health Research Institute, Barakaldo, Bizkaia, Spain; 17 Osakidetza, Cruces University Hospital, Barakaldo, Bizkaia, Spain; 18 Department of Internal Medicine, La Paz University Hospital, Madrid, Spain; 19 Working Group on Rare Diseases, Spanish Society of Internal Medicine (GTEM-SEMI), Madrid, Spain; 20 Internal medicine department - General Hospital Health Department, Alicante Institute for Health and Biomedical Research (ISABIAL), Alicante, Spain; 21 Department of Clinical Immunology, San Carlos University Clinical Hospital, Madrid, Spain; 22 Pediatric Infectious Diseases and Immunodeficiencies Unit, Hospital Universitari Vall d’Hebron, Vall d’Hebron Barcelona Hospital Campus, Barcelona, Catalonia, Spain; 23 Infection and Immunity in Pediatric Patients Research Group, Vall d’Hebron Institut de Recerca (VHIR), Vall d’Hebron Barcelona Hospital Campus, Barcelona, Catalonia, Spain; 24 Faculty of Medicine and Health Sciences, University of Barcelona, Barcelona, Spain; 25 Department of Internal Medicine, Lozano Blesa University Clinical Hospital, Zaragoza, Spain; 26 Aragon Health Research Institute, Zaragoza, Spain; 27 Working Group on Rare Diseases, Spanish Society of Internal Medicine, Zaragoza, Spain; 28 Endoscopy Unit, Dr. Balmis General University Hospital, Alicante, Spain; 29 Pediatric Allergy and Clinical Immunology Department, Clinical Immunology and Primary Immunodeficiencies Unit, Sant Joan de Déu Hospital, Barcelona, Spain; 30 Department of Surgery and Surgical Specialties, Faculty of Medicine and Health Sciences, University of Barcelona, Barcelona, Spain; 31 Study Group for Immune Dysfunction Diseases in Children (GEMDIP), Sant Joan de Déu Research Institute (IRSJD), Barcelona, Spain

**Keywords:** common variable immunodeficiency, Delphi technique, mass screening, neoplasms, population surveillance

## Abstract

**Introduction:**

Common variable immunodeficiency (CVID) is the most frequent symptomatic inborn error of immunity (IEI) in adults and is characterized by recurrent infections, immune dysregulation, and an increased risk of malignancy. Cancer represents a major cause of morbidity and mortality in this population, particularly hematological malignancies and gastric carcinoma. Data from the Spanish cohort report malignancy rates of up to 15%, with substantial variability in surveillance practices across centers. Despite this well-established cancer predisposition, standardized, evidence-based screening strategies are lacking, and no specific recommendations for malignancy surveillance in CVID currently exist. Therefore, this two-round Delphi consensus aimed to develop expert-based recommendations for cancer screening and surveillance in patients with CVID.

**Methods:**

The process included 39 multidisciplinary experts from 22 Spanish hospitals, including adult and pediatric centers, who evaluated a structured questionnaire comprising 87 statements across four domains: epidemiology/risk factors, solid tumors, hematological malignancies, and immunological biomarkers. The questionnaire was developed from a targeted literature review, and statements were rated using a five-point Likert scale, with consensus defined as ≥70% agreement/disagreement.

**Results:**

Overall, 70 statements, corresponding to 80.5%, reached agreement. Consensus supported systematic genetic testing at diagnosis and annual immunophenotyping to identify malignancy risk markers. In adults, baseline upper gastrointestinal endoscopy with histology-guided follow-up was recommended, together with annual *Helicobacter pylori* testing and monitoring of micronutrients and pernicious anemia-related antibodies. Hematological surveillance should include systematic evaluation of lymphadenopathy, with imaging ± biopsy when indicated, and bone marrow aspirate/biopsy in persistent (>6 months) cytopenias, while unexplained weight loss should prompt computed tomography imaging. Pediatric management should remain individualized and age-adapted, avoiding direct extrapolation from adult screening strategies.

**Discussion:**

This Delphi consensus proposes a structured approach to malignancy screening and surveillance in CVID from the time of diagnosis, encompassing gastric, hematological, and immunological assessment. These cancer-focused recommendations are intended to complement existing CVID management guidance, supporting earlier detection of malignancy and reducing variability in clinical practice.

## 1 Introduction

Primary antibody deficiencies (PAD) represent the most prevalent category and subtype of inborn errors of immunity (IEI), as classified by the International Union of Immunological Societies (IUIS) (1), and are associated with an increased risk of malignancy (1, 2). PAD was reported in 59.1% of all patients with cancer in the European Society for Immunodeficiencies (ESID) registry, and Common Variable Immunodeficiency (CVID), the most common symptomatic IEI in adults, accounted for 33.3% (2), with an estimated prevalence ranging from 1 in 10, 000 to 1 in 50, 000 individuals (3). Although the clinical spectrum of CVID is broad, ESID defines it as a PAD characterized by markedly reduced serum IgG and IgA levels, poor antibody responses to vaccination or low class-switched memory B cells, after exclusion of other secondary causes of hypogammaglobulinemia. Clinically, CVID presents with increased susceptibility to infections, most commonly of the respiratory and gastrointestinal tract, together with immune dysregulation manifestations such as autoimmunity, granulomatous disease, polyclonal lymphoproliferation, enteropathy, and an increased risk of malignancy (4).

Immunoglobulin replacement therapy (IgRT) limits infectious events in antibody-deficient patients. However, non-infectious complications are not controlled by this approach and constitute the leading cause of long-term morbidity and mortality in CVID (5, 6). In fact, the development of neoplasia in CVID has been associated with an age-standardized annual mortality rate 1.7-fold higher than in the general population and approximately threefold higher years of life lost due to premature mortality (7). Solid tumors and lymphomas appear to be the main drivers of mortality, exceeding the mortality impact of other CVID-related manifestations such as enteropathy, bronchiectasis, and autoimmunity (7). In line with this increased disease burden, hematologic malignancies represent approximately 70% of those reported in patients with CVID, followed by digestive tumors, according to a recent meta-analysis (8).

Several factors have been identified as potential indicators of malignancy risk in patients with CVID, including immune dysregulation (9), a history of immunosuppressive therapy (10), family history of cancer, and chronic *Helicobacter pylori* infection particularly for gastric cancer development (9, 11, 12), among others. Nevertheless, despite growing evidence supporting these associations, specific recommendations for cancer screening in CVID patients are still lacking (13). Existing consensus statements have mainly focused on the management of non-infectious complications, especially those related to immune dysregulation (14), while oncologic surveillance has received limited attention. Consequently, approaches to cancer screening in patients with CVID remain heterogeneous, with notable variability in the type and frequency of procedures implemented across centers. This gap is further illustrated by a previous publication based on a Spanish CVID cohort, which reported one of the highest neoplasia prevalences described to date, affecting approximately 15% of patients, together with marked heterogeneity in clinical practice (13).

In this context, the Working Group on Rare Diseases of the Spanish Society of Internal Medicine (GTEM-SEMI) initiated a Delphi consensus to develop expert-based recommendations for malignancy screening and surveillance in patients with CVID. This initiative aimed to harmonize current clinical practices and provide a standardized framework to guide the detection and follow-up of malignancies in both adult and pediatric patients with CVID. Importantly, these recommendations should be interpreted in an age-specific manner, as malignancy risk, tumor spectrum, and surveillance needs differ between adult and pediatric patients. They are also intended to complement previously established international consensus recommendations (14, 15) and national guidelines developed by the Spanish Primary Immunodeficiency Group for the diagnosis and management of patients with primary immunodeficiencies (16).

## 2 Material and methods

### 2.1 Literature search and review

A targeted literature review was performed to identify publications addressing malignancies in CVID, with emphasis on epidemiology, risk factors, immunologic biomarkers, and screening or surveillance strategies. Searches were conducted in PubMed/MEDLINE, Embase, Scopus, and Web of Science. Additional sources included reference lists of relevant articles and grey literature, such as consensus statements and guidelines from professional societies.

The search strategy combined controlled vocabulary and free-text terms related to CVID and malignancy, including “common variable immunodeficiency” OR “CVID” AND “cancer” OR “malignancy” OR “neoplasm” OR “lymphoma” OR “gastric cancer” OR “screening” OR “surveillance” OR “risk factor*” OR “biomarker*”. Filters were applied when appropriate to retrieve clinical guidelines, cohort studies, registry analyses, and systematic reviews.

The evidence was narratively synthesized according to four main thematic domains: prevalence and risk factors, hematological malignancies, gastrointestinal and other solid tumors, and immune dysregulation with associated biomarkers. Discrepancies in study inclusion or interpretation were resolved through discussion within the Scientific Committee.

The body of evidence retrieved through this process served as the foundation for developing and refining the statements subsequently evaluated in the Delphi consensus.

### 2.2 Development of consensus statements

A Delphi consensus methodology (17) was employed to develop expert-based recommendations on malignancy screening in patients with CVID. The process was coordinated by the GTEM-SEMI between April and September 2025 and involved two groups: a Scientific Committee and a Panel of Experts.

The Scientific Committee comprised 20 specialists from reference centers across Spain, including experts in Internal Medicine, Immunology, Hematology, Oncology, Gastroenterology, and Pediatrics. Its members defined the scope of the consensus, drafted the initial set of statements, and reviewed the final recommendations. The same specialists also participated in the Delphi rounds as respondents, ensuring alignment between statement formulation and evaluation.

The Panel of Experts included clinicians from the same specialties, selected for their experience in the management of IEI and related malignancies. Panel members independently and anonymously evaluated all statements through two sequential online survey rounds.

Each statement was rated using a five-point Likert scale (1 = strongly disagree, 2 = disagree, 3 = partially agree, 4 = agree, 5 = strongly agree). A predefined threshold of ≥70% agreement (sum of “agree” and “strongly agree”) or ≥70% disagreement (sum of “disagree” and “strongly disagree”) was required to establish consensus. Statements not reaching consensus after the first round were revised based on participants’ comments and re-circulated in the second round.

Descriptive analyses were used to calculate the percentage of agreement for each statement. Items achieving consensus were summarized as final recommendations, while those that did not were retained for further discussion.

Following completion of both Delphi rounds, a hybrid consensus meeting was held with all members of the Scientific Committee. During this meeting, results were reviewed, unresolved items were discussed, and the key aspects for manuscript drafting were agreed upon. Free-text comments from respondents were considered in refining the recommendations and structuring the Discussion section of this manuscript.

## 3 Results

### 3.1 Participants

The Delphi process included 39 specialists from 22 hospitals across different Autonomous Communities in Spain, ensuring broad geographical and institutional representation. Participants primarily belonged to Internal Medicine (33.3%), Pediatrics (23.1%), and Immunology (15.4%), together with experts in Gastroenterology, Hematology, Oncology, and Infectious Diseases. Most respondents (69.2%) managed exclusively adult patients, 20.5% treated pediatric patients, and 10.3% cared for both populations. More than half of the panel (51.3%) reported over 15 years of clinical experience in IEI management, reflecting a highly experienced expert group.

Of the 39 specialists, 30 provided additional information on the clinical profile of patients under follow-up at their institutions. After consolidating responses from specialists belonging to the same center, data from 19 hospitals were available.

Based on the information collected through the Delphi survey, patient data on CVID were available from 30 respondents, corresponding to 19 hospitals across 6 different autonomous communities after consolidating answers from specialists belonging to the same center. Across these hospitals, the mean number of CVID patients under follow-up was 105.1 per hospital, with reported values ranging from 10 to 169 patients. Using these consolidated data, the estimated number of CVID patients across the participating hospitals was approximately 1997. When related to the Spanish population of 49 442–844 inhabitants as of October 1, 2025 (18), this figure corresponds to an approximate prevalence of 40.4 cases per million inhabitants. Since the survey included hospitals that participated voluntarily and did not represent all centers managing CVID in Spain, this prevalence should be interpreted strictly as an approximation derived from the available dataset.

### 3.2 Consensus overview

The Delphi survey included 87 statements grouped into four thematic areas: epidemiology and risk factors for malignancy in CVID, hematological malignancies, solid tumors and gastrointestinal cancer screening, and immune dysregulation and immunological biomarkers.

Of the 87 statements, 70 (80.5%) reached consensus in agreement, 1 (1.1%) reached consensus in disagreement, and 16 (18.4%) did not reach consensus after two rounds. The agreed statements formed the basis for the final recommendations. The remaining items were retained for discussion, as they reflected areas of uncertainty or insufficient evidence in the literature.

### 3.3 Epidemiology and risk factors for malignancy in CVID

Consensus was reached for multiple items identifying the prevalence and major determinants of malignancy in CVID (**Table 1**). The experts agreed that approximately 10% of patients with CVID develop some form of neoplasm, and that CVID represents the IEI most frequently associated with cancer development, with no pathogenic genetic mutation identified in the majority of cases. Hematological malignancies were identified as the most common neoplasia, followed by gastric cancer (100% agreement). The panel further agreed that patients developing lymphoid malignancies presented the highest mortality.

**Table 1:** Prevalence and risk factors.

Statement	Result (%)
Approximately 10% of patients with CVID develop some type of neoplasm.	89.74
CVID is the IEI without a known pathogenic variant, most frequently associated with the development of malignant neoplasms.	87.18
Among patients with CVID, hematological malignancies are the most frequently diagnosed, followed by gastric cancers.	100
Patients with a cancer phenotype (particularly lymphoid malignancies) have the highest mortality rate.	79.49
Periodic screening protocols are necessary in these patients to enable early diagnosis of malignancies due to their increased risk.	87.18
CVID patients aged over 40 years require closer monitoring due to higher malignancy risk.	97.44
Factors potentially associated with malignancy development in CVID include male sex, smoking, low socioeconomic status, gastric surgery, and consumption of processed foods.	94.87
Older age at onset or diagnosis of immunodeficiency.	66.67
Family history of cancer.	87.18
Presence of immune dysregulation (celiac disease, inflammatory bowel disease, systemic lupus erythematosus, Sjögren’s syndrome, rheumatoid arthritis, psoriasis, autoimmune cytopenias).	97.44
History of immunosuppressive therapy.	79.49
Elevated IgM levels.	53.85
Lower CD4+ lymphocyte count at diagnosis.	89.74
Lower CD8+ lymphocyte count at diagnosis.	51.28
Low vitamin B12 levels.	41.03
Pernicious anemia, defined as a megaloblastic anemia caused by vitamin B12 deficiency secondary to autoimmune gastritis and characterized by the presence of anti–IF and/or anti–GPC antibodies, as a risk factor for precancerous gastric lesions.	89.74
Atrophic gastritis as a risk factor for precancerous gastric lesions.	94.87
Absolute or markedly reduced IgA levels as a risk factor for precancerous gastric lesions.	61.54
Chronic *H. pylori* infection as a risk factor for precancerous gastric lesions.	94.87
The most frequent cause of gastric neoplasms in CVID is autoimmune in nature.	41.03
The most frequent cause of gastric neoplasms in CVID is *H. pylori* infection.	84.62

Dark green and dark red indicate consensus on agreement and disagreement, respectively. Light green and light red indicate no consensus and reflect the predominant response direction.

Building upon these findings, consensus also supported the implementation of periodic screening protocols to facilitate early malignancy detection. Experts highlighted that patients aged over 40 years required closer monitoring due to their increased cancer risk (97.44% agreement). Similarly, several general demographic and lifestyle factors beyond the underlying immune deficiency were identified as potential contributors to malignancy risk, as observed in the general population, including male sex, smoking, low socioeconomic status, prior gastric surgery, and consumption of processed foods.

In addition to these parameters, other factors achieved consensus as relevant risk indicators. These included a family history of cancer, diagnosis of celiac disease, the development of immune dysregulation manifestations such as autoimmune cytopenias, inflammatory bowel disease, systemic lupus erythematosus, Sjögren’s syndrome, rheumatoid arthritis, or psoriasis, and history of prior immunosuppressive therapy.

The panel also assessed immunological and clinical factors associated with malignancy in CVID, with relevance varying by cancer type.

Overall, lower CD4+ lymphocyte counts at diagnosis were linked to an increased overall risk of malignancy. Particularly in the context of gastric carcinogenesis, risk factors clustered around chronic mucosal inflammation and autoimmune gastric disease. Pernicious anemia, defined as a megaloblastic anemia caused by vitamin B12 deficiency secondary to autoimmune gastritis and characterized by the presence of anti–intrinsic factor (anti-IF) and/or anti–gastric parietal cell (anti-GPC) antibodies, together with atrophic gastritis were considered predisposing factors for precancerous gastric lesions. Vitamin B12 also had two distinct clinical implications. On the one hand, low vitamin B12 levels may raise suspicion of pernicious anemia diagnosis and, therefore, of increased gastric cancer risk. On the other hand, elevated vitamin B12 levels may serve as a potential indicator of an underlying lymphoproliferative disorder. Chronic *H. pylori* infection was strongly endorsed as a major etiological determinant (94.87% agreement), with consensus that it represents the most frequent cause of gastric neoplasms in CVID. In contrast, no agreement was reached on an autoimmune origin as the predominant mechanism (20.51% agreement, 38.46% partial, 41.03% disagreement), underscoring the complexity of disentangling immune-mediated and infection-driven pathways in gastric carcinogenesis in this population.

Despite these areas of agreement, several proposed risk factors failed to reach consensus, reflecting ongoing uncertainty in specific areas. These included older age at onset or diagnosis (66.67% agreement, 23.08% partial, 10.26% disagreement), elevated IgM levels (53.85% agreement, 43.59% partial, 2.56% disagreement), lower CD8+ lymphocyte counts (51.28% agreement), and absolute or markedly reduced IgA levels as a risk factor itself (61.54% agreement).

### 3.4 Solid tumors screening

Consensus was reached for most items addressing the management and prevention of solid tumors in CVID (**Table 2**). Panelists were asked about viruses with an oncogenic role in CVID. Screening for Epstein–Barr virus (EBV) and herpes simplex virus (HSV) did not reach consensus (64.0% and 4.0% agreement, respectively), whereas screening for human papillomavirus (HPV) was recommended (80.8% agreement). However, no consensus was achieved regarding the optimal screening interval; therefore, patients should at least be included in standard population-based HPV screening programs. The panel also acknowledged that more than half of adult CVID patients experience *H. pylori* recurrence after eradication, underscoring the need for long-term surveillance, although no consensus was reached either regarding the optimal frequency for performing this screening. In addition, it is important to highlight that unexplained weight loss was regarded as an indication for full-body computed tomography (CT) evaluation, given its potential to reflect underlying malignancy especially in this at-risk population.

**Table 2:** Screening of solid malignancies.

Statement	Result (%)
Screening for Epstein–Barr virus (EBV) infection is recommended, as it increases susceptibility to malignancies.	64.00
Screening for herpes simplex virus (HSV) infection is recommended, as it increases susceptibility to malignancies.	68.00
Screening for human papillomavirus (HPV) infection is recommended, as it increases susceptibility to malignancies.	80.77
Upper gastrointestinal endoscopy should be performed at the time of CVID diagnosis in adults, together with *H. pylori* testing and eradication therapy even in asymptomatic patients.	82.76
In CVID patients with gastrointestinal symptoms, endoscopy with biopsy should be performed to detect *H. pylori* infection.	96.55
More than half of adult CVID patients who eradicated *H. pylori* experience recurrence.	86.21
The screening protocol for selecting CVID patients at higher risk of gastric cancer should include simple, non-invasive annual tests such as UBT or stool antigen test for *H. pylori*, vitamin B12, IF/GPC antibodies, and serum iron profile.	75.86
Pernicious anemia, atrophic gastritis, low IgA, and chronic *H. pylori* infection are the main risk factors for developing precancerous gastric lesions.	96.55
Unexplained weight loss in a CVID patient warrants a full-body CT scan, among other tests.	100
Patients with premalignant gastric lesions should undergo endoscopic surveillance every 6 months for high-grade dysplasia or every 12 months for low-grade dysplasia.	93.10
Close clinical follow-up is recommended for patients with CVID who present with intestinal villous atrophy, as this subgroup shows significantly increased mortality, mainly driven by oncohematologic and infectious complications.	79.31
In the absence of premalignant findings, gastroscopy should be repeated at least every 3–5 years.	75.86
In CVID patients with pernicious anemia, follow-up endoscopy is not recommended when histopathology is normal or shows isolated antral atrophy.	72.41
In CVID patients with pernicious anemia, repeating endoscopy in 5 years is recommended for chronic gastritis.	75.86
In CVID patients with pernicious anemia, repeating endoscopy in 3 years is recommended for advanced atrophic gastritis or intestinal metaplasia in the antrum and corpus; in the presence of a family history of gastric cancer, it should be performed every 1–2 years.	75.86
In CVID patients with pernicious anemia, repeating endoscopy in 6 months is recommended if there is high-grade dysplasia, or at 12 months for low-grade dysplasia.	96.55
Upper gastrointestinal endoscopy should be performed in patients with low B12 levels, positive UBT, dyspepsia, or unexplained weight loss.	93.10
High-quality upper endoscopy for detecting premalignant and malignant gastric lesions should use high-definition white-light endoscopy.	89.66
High-definition chromoendoscopy is superior to standard white-light endoscopy for diagnosing precancerous gastric conditions and early neoplasms.	72.41
For gastric cancer screening, biopsies should be taken from at least two topographic sites (antrum and corpus, along both curvatures).	96.55
Systematic gastric biopsies are recommended in all CVID patients, even in the absence of macroscopic lesions, to allow early detection of malignant progression.	79.31
CVID patients with malabsorption should be closely monitored, as neoplasms are more frequent in this group.	86.21
Reduced plasma cell density in gastric mucosa tissue may serve as a marker of a “more inflammatory phenotype” and low-grade immune activation.	62.07
In CVID patients with celiac-like manifestations, upper gastrointestinal endoscopy and biopsies should be performed before initiating a gluten-free diet.	93.10
HLA (DQ2, DQ8) testing should be included in the screening of CVID patients with villous atrophy to differentiate celiac disease from other causes and establish malignancy risk.	93.10
Because breast cancer is among the most frequent malignancies in CVID, mammography screening should begin earlier and be performed more frequently than in the general population.	65.52
Increased frequency of fecal occult blood testing and colonoscopy is recommended for colorectal cancer screening in CVID patients.	72.00

Dark green and dark red indicate consensus on agreement and disagreement, respectively. Light green and light red indicate no consensus and reflect the predominant response direction.

**Figure 1:**
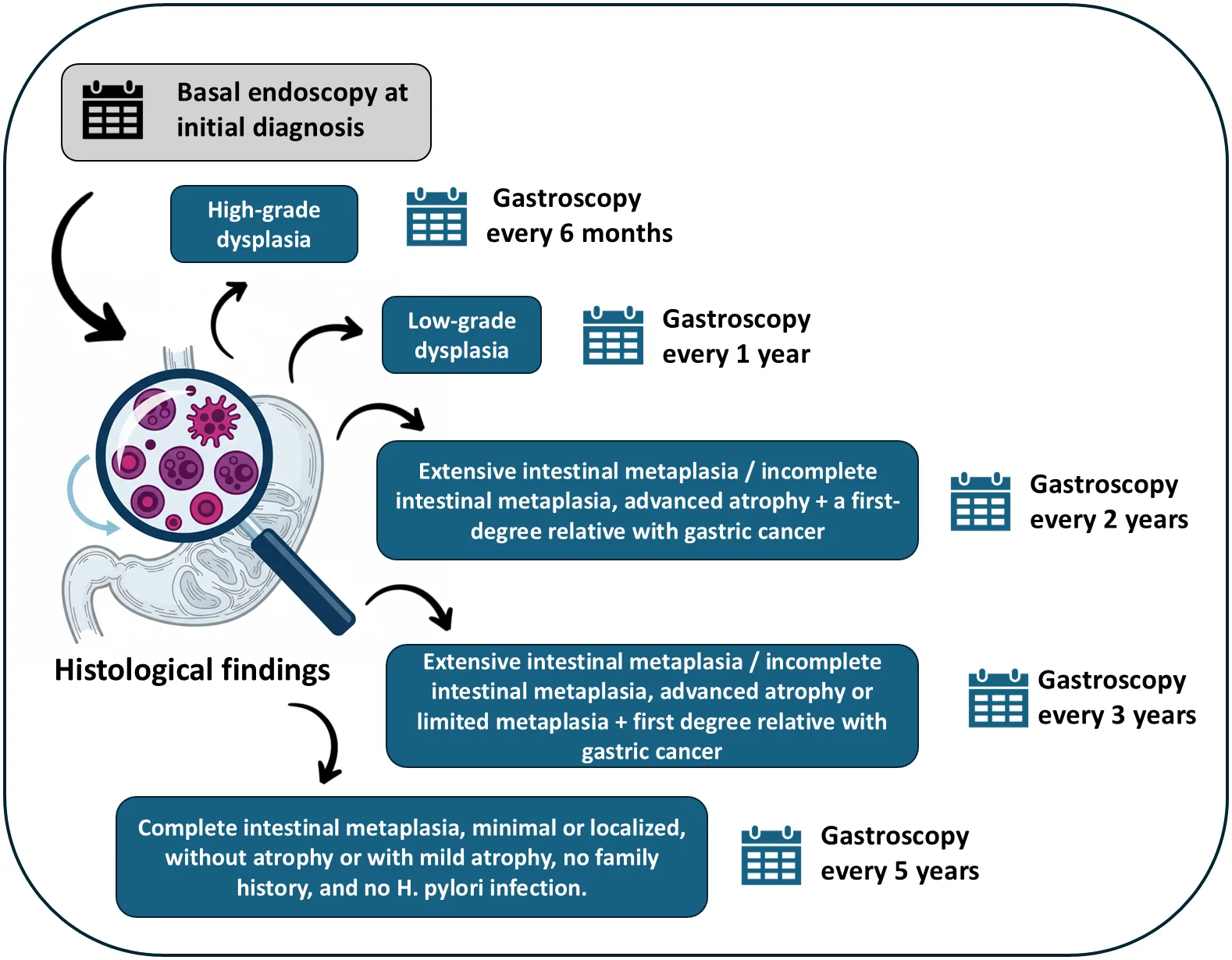
Recommended frequency of endoscopic surveillance in adult patients. Figure based on the European MAPS II digestive endoscopy guidelines (20). H. pylori, Helicobacter pylori.

Within this broader preventive approach, gastric cancer screening was addressed in detail. At CVID diagnosis in adults, the panel supported performing upper gastrointestinal endoscopy with gastric biopsies following Sydney protocol (19) in all patients, including asymptomatic individuals, with *H. pylori* identification performed on the gastric biopsy sample itself and eradication therapy if positive (82.76% agreement). Additionally, limited to adults, baseline assessment should include determining serum vitamin B12, iron profile, and anti-IF and anti-GPC antibodies. During follow-up, adult patients should undergo annual non-invasive *H. pylori* screening using either a urea breath test (UBT) or stool antigen test, together with periodic evaluation of serum vitamin B12 levels, anti-intrinsic factor antibodies, anti-parietal cell antibodies, and iron profile. To operationalize follow-up once gastric precancerous lesions are identified, and although this item was not assessed in the Delphi consensus, the scientific committee recommended endoscopic surveillance in line with the latest European guidelines for the management of gastric precancerous lesions (20), with follow-up intervals ranging from six months to five years according to histological grade and risk as outlined in **Figure 1**.

Notably, CVID patients presenting with malabsorption were identified as a high-risk subgroup requiring close clinical monitoring (86.21% agreement).

In patients with pernicious anemia, follow-up intervals were also defined according to histopathological findings. There was disagreement with omitting follow-up when histopathology is normal or shows isolated antral atrophy (72.41% disagreement). Repeating endoscopy in 5 years for chronic gastritis (75.86% agreement), in 3 years for advanced atrophic gastritis or intestinal metaplasia (75.86% agreement), and at 12 months for low-grade dysplasia or 6 months for high-grade dysplasia (96.55% agreement) was recommended. Upper endoscopy was also recommended for patients with low vitamin B12 levels, positive UBT, dyspepsia, or unexplained weight loss (93.10% agreement).

High-definition white-light endoscopy was considered the preferred method for detecting premalignant and malignant lesions (89.66% agreement), and high-definition chromoendoscopy was regarded as superior to standard white-light endoscopy for diagnosing precancerous gastric conditions and early neoplasms (72.41% agreement). Biopsies should be obtained from at least two topographic sites (antrum and corpus) along both curvatures (96.55% agreement), and systematic biopsies were recommended in all CVID patients following Sydney protocol (19), even in the absence of macroscopic abnormalities (79.31% agreement).

Consensus was also reached that patients with celiac-like manifestations should undergo upper gastrointestinal endoscopy and biopsy before initiating a gluten-free diet (93.10% agreement). Human leukocyte antigen (HLA)-DQ2/DQ8 testing should be performed to distinguish celiac disease from other causes of villous atrophy and to help establish malignancy risk (93.10% agreement).

Regarding the screening of other solid tumors in adults, the panel recommended increasing the frequency of fecal occult blood testing and colonoscopy for colorectal cancer surveillance (72.0% agreement). However, no consensus was reached on the optimal surveillance interval, largely due to the limited available scientific evidence supporting specific screening periodicities in this context.

### 3.5 Hematological malignancies

Consensus was achieved for all major statements addressing hematological malignancies in CVID (**Table 3**). The panel agreed that the most frequently diagnosed lymphomas are non-Hodgkin B-cell lymphomas. Experts also concurred that patients presenting with hematological cancers of unclear origin should be screened for primary immunodeficiencies to identify potential underlying CVID (96.55% agreement).

**Table 3:** Screening of hematological malignancies.

Statement	Result (%)
The most frequently diagnosed lymphomas in CVID are non-Hodgkin B-cell lymphomas, particularly extranodal mucosa-associated lymphoid tissue, marginal zone, and Epstein–Barr virus–associated lymphomas.	100
It is important to screen patients with secondary immunodeficiencies in the context of hematological cancer, as they may conceal an underlying IEI.	96.55
In the presence of lymphadenopathy in a CVID patient, ultrasound evaluation and biopsy should be performed.	86.21
Presence of lymphadenopathy may indicate suspected hematological malignancy in CVID patients.	96.55
Elevated vitamin B12 levels may indicate suspected hematological malignancy in CVID patients.	85.71
Elevated beta-2 microglobulin may indicate suspected hematological malignancy in CVID patients.	79.31
Presence of cytopenias may indicate suspected hematological malignancy in CVID patients.	89.66
Presence of a monoclonal peak in serum protein electrophoresis may indicate suspected hematological malignancy in CVID patients.	82.76
Elevated LDH may indicate suspected hematological malignancy in CVID patients.	72.41
Altered kappa/lambda light-chain ratio may indicate suspected hematological malignancy in CVID patients.	75.86
In patients with IEI and persistent (>6 months) or refractory cytopenias, bone marrow biopsy should be performed to rule out myelodysplastic or lymphoproliferative syndromes.	82.76
In patients with IEI and persistent (>6 months) or refractory cytopenias, bone marrow aspiration should be performed to rule out myelodysplastic or lymphoproliferative syndromes.	86.21
Early-onset CVID is associated with greater immune dysregulation in pediatric patients.	85.71
Non-Hodgkin lymphomas are the most frequent tumors in pediatric patients with CVID.	85.71
In pediatric patients, the most frequent cause of localized lymphoid neoplasia is Hodgkin lymphoma, typically diagnosed during adolescence.	85.71
Although Hodgkin lymphoma commonly presents with lateral cervical lymphadenopathy, axillary and inguinal nodes should also be examined.	85.71
Children with inborn errors of immunity have increased susceptibility to Epstein–Barr virus infection and a higher risk of malignant clonal lymphoproliferation.	71.43
Stratification criteria used in adult CVID patients cannot be directly extrapolated to pediatric cases due to ongoing immune maturation; pediatric-specific criteria should be developed and complemented by longitudinal assessments.	100
Lymph node biopsy should be performed during the initial diagnostic evaluation in children with persistent lymphoproliferation.	71.43
Oncology evaluation should start with ultrasound, and if suspicious findings (e.g., loss of hilar structure) are present, PET-CT should be used to identify the node with highest uptake for diagnostic biopsy.	71.43
Genetic testing should be performed in all pediatric CVID patients at diagnosis, especially in those with immune dysregulation.	100

Dark green and dark red indicate consensus on agreement and disagreement, respectively. Light green and light red indicate no consensus and reflect the predominant response direction.

Regarding diagnostic evaluation, consensus was reached that the presence of lymphadenopathies on physical examination in adult patients with CVID warrants ultrasound assessment and considering biopsy, given its potential association with hematological malignancy. Similarly, unexplained weight loss was regarded as an indication for full-body CT evaluation, given its potential to reflect underlying malignancy.

Several laboratory markers were recognized as potential indicators of hematologic malignancy, including elevated vitamin B12, beta-2 microglobulin, lactate dehydrogenase (LDH), and an altered kappa/lambda light-chain ratio. The presence of cytopenias (89.66% agreement) or a monoclonal peak on serum protein electrophoresis (82.76%) was likewise regarded as a suspicious finding requiring further evaluation. In addition, agreement was achieved that, in patients with CVID and persistent (over six months) or treatment-refractory cytopenias, both bone marrow aspiration (86.21%) and biopsy (82.76% agreement) should be performed to rule out myelodysplastic or lymphoproliferative syndromes.

Pediatric-specific items similarly reached consensus. The panel agreed that stratification criteria used in adults cannot be directly extrapolated to pediatric cases and that specific criteria should be developed (100%). Early-onset CVID was associated with greater immune dysregulation (85.71% agreement), and non-Hodgkin lymphomas were identified as the most frequent tumors in pediatric patients (85.71%). Children with CVID were recognized to have increased susceptibility to EBV infection and a higher risk of malignant clonal lymphoproliferation (71.43% agreement). In the presence of lymphadenopathy, ultrasound was recommended as the first-line imaging modality, with positron emission tomography (PET)–CT indicated in cases with suspicious findings (71.4% agreement). For persistent lymphoproliferation, lymph node biopsy was recommended as part of the initial diagnostic evaluation (71.4% agreement). Finally, as in adults, genetic testing was recommended for all pediatric CVID patients, particularly those presenting with immune dysregulation (100% agreement).

### 3.6 Immune dysregulation and immunological biomarkers

Consensus was achieved for nearly all statements addressing immune dysregulation and the use of certain immunological biomarkers for cancer monitoring in CVID (**Table 4**). Genetic testing was recommended for all patients at the time of diagnosis, particularly for those presenting with features of immune dysregulation (72.00% agreement). Several genetic variants, including *PIK3CD*, *PIK3R1*, *CD27*, and *CD70*, were recognized as conferring a significantly higher risk of lymphomagenesis, underscoring the importance of their identification for accurate diagnosis and optimal management.

**Table 4:** Immune dysregulation and biomarkers.

Statement	Result (%)
Genetic testing is recommended for all CVID patients at the time of diagnosis, particularly those presenting with immune dysregulation.	72.00
Suspicion of malignancy in CVID patients may arise in the presence of elevated IgM levels.	53.85
Suspicion of malignancy in CVID patients may arise in the presence of elevated CD8+ T-cell counts.	51.28
Suspicion of malignancy in CVID patients may arise in the presence of unexplained elevated IgG levels.	55.17
Suspicion of malignancy in CVID patients may arise in the presence of elevated IgA levels.	51.72
Given that immune dysregulation in CVID is associated with an increased cancer risk, genetic screening for cancer-associated genes should be performed to assess patient risk.	55.17
Close monitoring is recommended in CVID patients presenting with reduced regulatory T cells (Tregs).	73.33
Close monitoring is recommended in CVID patients presenting with increased follicular helper T cells.	62.07
Close monitoring is recommended in CVID patients presenting with reduced class-switched memory B cells.	75.86
Close monitoring is recommended in CVID patients presenting with increased CD21low B cells.	83.33
Some genetic variants, such as PIK3CD, PIK3R1, CD27, or CD70 confer a significantly higher risk of lymphomagenesis in CVID, making their identification crucial for diagnosis and management.*	93.33
Expansion of atypical B cells (ABCs), which can represent up to 30% of the CD21low B-cell population in CVID (versus 5% in healthy controls), may serve as a key biomarker for malignancy risk and immune dysregulation.	80.00
Flow cytometric immunophenotyping of lymphocyte subpopulations should be performed at least once per year to detect abnormalities indicative of immune dysregulation or lymphoproliferation.	83.33

Dark green and dark red indicate consensus on agreement and disagreement, respectively. Light green and light red indicate no consensus and reflect the predominant response direction.

*The genes listed in this Delphi statement were included as examples and should not be interpreted as an exhaustive list of variants associated with lymphoproliferation or lymphomagenesis in CVID.

Additionally, close clinical and immunological monitoring was advised in patients showing reduced regulatory T cells (Tregs), reduced class-switched memory B cells, or increased CD21^low B cells. Expansion of atypical B cells (ABCs), also termed age-associated B cells and commonly identified within the CD21low B-cell compartment in CVID, was highlighted as a key biomarker for malignancy risk and immune dysregulation (80.00% agreement). Phenotypically, these cells differ from conventional B cells by the expression of the T-cell-associated transcription factor T-bet and the myeloid surface marker CD11c. In CVID, CD21low B-cell expansion has been associated with non-infectious complications and autoimmune dysregulation, potentially reflecting pathological chronic stimulation in patients lacking normal antibody-production pathways (21, 22). Finally, consensus was reached that flow cytometric immunophenotyping of lymphocyte subpopulations should be performed at least once per year to identify abnormalities indicative of immune dysregulation or emerging lymphoproliferation (83.33% agreement).

## 4 Discussion

This Delphi consensus highlights the increasing recognition of cancer as a major non-infectious complication of CVID, which entails the highest mortality among all non-infectious manifestations, and represents a coordinated effort to harmonize clinical practice regarding screening and surveillance strategies. Despite the heterogeneity of available evidence, a broad multidisciplinary agreement emerged on the need for structured national cancer screening. By integrating perspectives from multiple specialties, this initiative provides an expert-based framework intended to reduce variability across centers and to incorporate malignancy prevention and early detection protocols into routine CVID management.

Among the issues addressed, the panel evaluated age at disease onset as a potential modifier of malignancy risk; however, no consensus was reached on its role as an independent determinant. Divergent perspectives emerged, with adult clinicians linking older age at diagnosis to prolonged diagnostic delay and sustained immune dysregulation, and pediatric clinicians emphasizing the more severe, inflammatory phenotypes associated with early-onset disease (10, 23–25). Overall, age should be interpreted within a broader clinical and immunological context rather than as an isolated predictor of malignancy risk (10, 23–25). The panel emphasized that recognizing the typical malignancy spectrum in CVID is key to guiding clinical suspicion, with hematological and gastric cancers identified as the most frequent in adults, consistent with previous literature (9, 13, 26, 27). Importantly, this distribution should be interpreted in an adult-specific context. Gastric cancer is extremely uncommon in pediatric cohorts, where early-onset presentations more often reflect genetically defined IEI rather than classic CVID (28, 29). Consequently, throughout the consensus, statements addressing malignancy risk and cancer distribution were considered primarily applicable to adult CVID unless otherwise stated. This age-related distinction is not merely epidemiological but has direct practical consequences for follow-up, because pediatric diagnostic pathways should not represent a simple extrapolation of adult protocols. In children, surveillance should be driven more strongly by longitudinal clinical evolution, persistent lymphoproliferation, EBV-related risk, and the possibility of a genetically defined IEI phenotype (30, 31), whereas adult protocols more commonly prioritize gastric premalignant lesions and other age-associated risks (12, 32). However, this age-specific approach does not preclude standard, age-based population screening, which remains recommended for all CVID patients, children and adults alike, alongside the CVID-specific surveillance measures proposed here.

Therefore, gastric cancer prevention and surveillance emerged as a key focus, driven by the link between chronic mucosal inflammation and carcinogenesis. Pernicious anemia, chronic *H. pylori* infection and atrophic gastritis were identified as major risk factors for precancerous lesions. Accordingly, baseline upper endoscopy with systematic biopsies following Sydney protocol (19) at CVID diagnosis, including *H. pylori* assessment and eradication if positive, was recommended as a preventive strategy in all adult patients (12, 13, 20, 33, 34). However, although agreement was reached on the need for endoscopic surveillance after baseline gastroscopy, no agreement was achieved on the follow-up gastroscopy interval, therefore the scientific committee recommends adhering, at minimum, to the surveillance frequency proposed in the European MAPS II digestive endoscopy guidelines (35). In addition, annual non-invasive monitoring, including either UBT or stool *H. pylori* antigen testing, vitamin B12, iron profile, anti-IF and anti-GPC antibodies, was unanimously endorsed as a pragmatic approach to identify early warning signs of malignant transformation. At the same time, the panel acknowledged persistent uncertainty regarding the clinical utility of immunological biomarkers to refine gastric cancer risk prediction. The predictive value of markers such as IgM, IgA, or CD8+ lymphocyte parameters remains unclear, as immune activation may reflect both compensatory responses and pathogenic immune dysregulation. Accordingly, the statement proposing that absolute or markedly reduced IgA levels confer increased risk of precancerous gastric lesions did not reach consensus, reflecting inconclusive and conflicting evidence, with earlier reports suggesting a role for IgA deficiency (9), and more recent data describing higher IgA levels among CVID patients who developed malignancies (26). These observations are not contradictory but reflect distinct pathophysiological entities: low IgA may impair mucosal secretory immunity and predispose to gastric carcinogenesis, whereas a relatively elevated IgA in the context of CVID should raise suspicion of an underlying lymphoproliferative disorder or plasma cell dyscrasia, in which IgA elevation may represent a direct consequence of the malignant process itself. Additionally, experts cautioned against empirical prescription of a gluten-free diet in CVID patients without histological confirmation of celiac disease, particularly given the associated increased risk of intestinal lymphoma (36, 37), as initiating dietary restriction before appropriate endoscopic evaluation may mask relevant histopathological findings and delay the diagnosis of neoplastic lesions (38).

Beyond gastric cancer, the panel also evaluated colorectal and breast cancer due to their high population incidence and established screening programs. Although an increased prevalence of breast cancer has been suggested in CVID, evidence remains limited and inconsistent (26), and less robust than for gastric or hematologic malignancies. The lack of prospective data precludes CVID-specific screening recommendations. Until further evidence is available, patients should be included in standard population-based breast and colorectal cancer screening programs. Regarding hematological malignancies, the consensus emphasized systematic assessment of lymphadenopathy on physical examination, with further evaluation of detected nodes by ultrasound and biopsy when indicated, as well as prompt investigation of persistent cytopenias, including aspiration and bone marrow biopsy. This approach was supported as a pragmatic strategy to exclude myelodysplastic syndromes and lymphoproliferative disorders, in line with registry-based evidence in CVID and related IEIs (6, 27, 39, 40). Notably, cytopenias may represent an early, and sometimes the only, indicator of underlying onco-hematological disease in a population where immune dysregulation may blur typical clinical and laboratory warning signs.

Immune dysregulation was consistently framed as a unifying background that contextualizes malignancy predisposition in CVID (10). The discussion generated within this consensus reflects an evolution in the understanding of CVID from a disorder defined primarily by antibody deficiency to a complex syndrome characterized by immune dysregulation and chronic inflammation with systemic implications (4, 41). This conceptual shift aligns with studies linking immune dysregulation, immune senescence, and specific genetic variants to neoplastic transformation in CVID (8, 10), acknowledging associations between variants in genes such as *NFKB1*, *CTLA4*, *LRBA*, *PIK3CD*, and *IKZF1* with immune dysregulation and lymphoid neoplasia (3, 42). In this context, experts also agreed on the value of annual immunophenotyping to support longitudinal identification of immune dysregulation phenotypes associated with increased oncologic risk and emphasized that establishing a monogenic cause should not exclude patients from structured follow-up, given the substantial clinical and pathophysiological overlap with classical CVID. Nevertheless, disparities in access to genetic testing persist, emphasizing the need for harmonized diagnostic pathways across healthcare systems. Taken together, these elements support the rationale for incorporating genetic testing at diagnosis, in line with what has been suggested by previously published evidence (42, 43).

**Figure 2:**
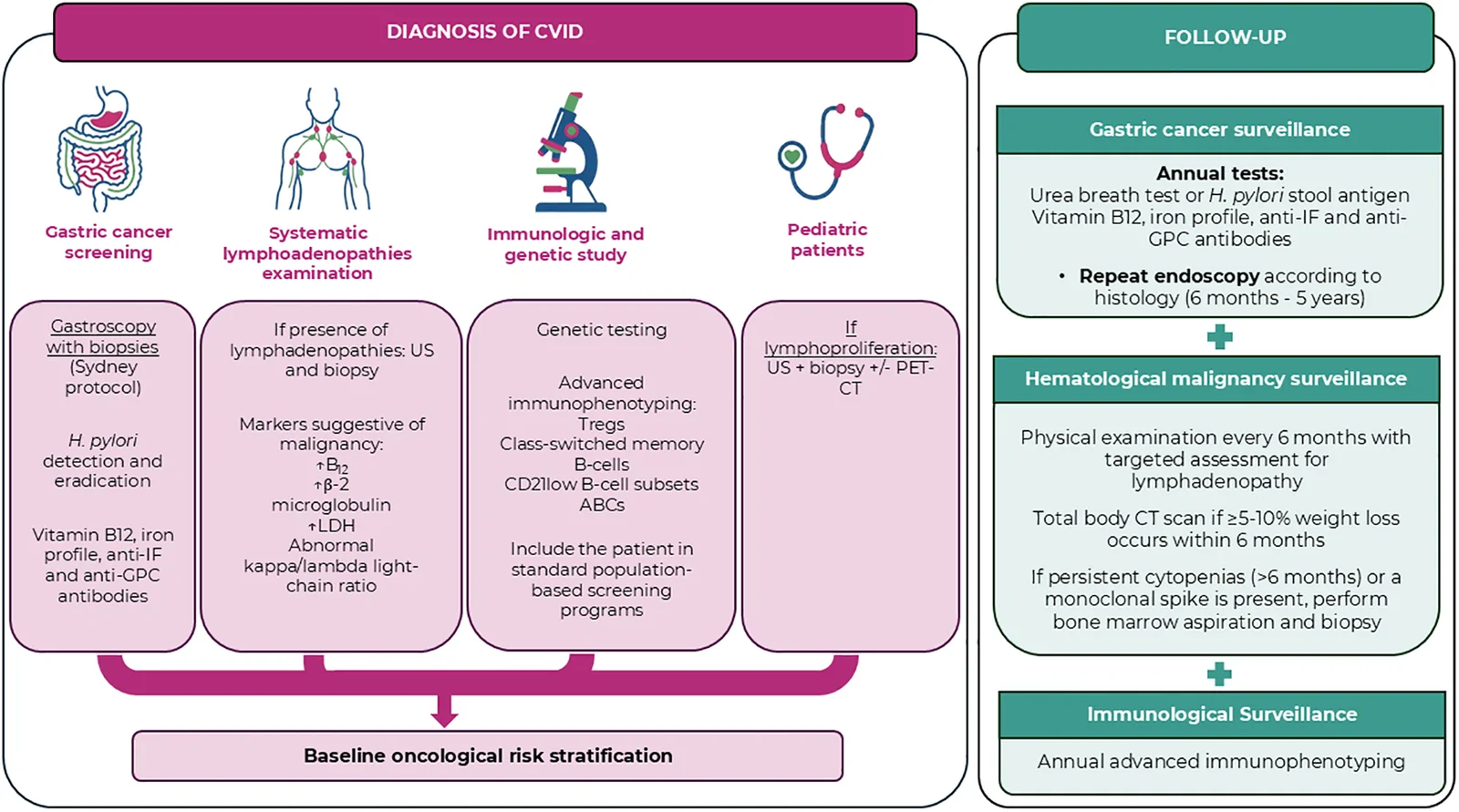
Consensus-based recommendations for diagnosis and follow-up of malignancies in patients with CVID. ABCs, atypical B cells; anti-GPC, anti-gastric parietal cell; anti-IF, anti-intrinsic factor; CT, computed tomography; CVID, common variable immunodeficiency; H. pylori, Helicobacter pylori; LDH, lactate dehydrogenase; PET-CT, positron emission tomography-computed tomography; Tregs, regulatory T cells; US, ultrasound.

Based on these considerations, this consensus proposes a structured, proactive, and individualized malignancy surveillance strategy as a core component of CVID management (**Figure 2**). Cancer screening should be integrated into routine follow-up from the time of diagnosis, with particular emphasis on malignancies with the strongest and most consistent risk, namely gastric and hematological cancers (26).

At CVID diagnosis, baseline oncological risk stratification should include: (i) upper gastrointestinal endoscopy with systematic biopsies according to the Sydney protocol, including assessment for *H. pylori* and eradication if positive, together with evaluation of serum vitamin B12, iron profile, anti-intrinsic factor, and anti-parietal cell antibodies; (ii) Comprehensive assessment of lymphadenopathy on physical examination, with ultrasound evaluation and biopsy when indicated, together with evaluation of malignancy-associated biomarkers (elevated vitamin B12, β2-microglobulin, LDH, and abnormal κ/λ light chain ratio); and (iii) laboratory evaluation including genetic testing and advanced immunophenotyping, defined as extended flow cytometric panels beyond basic lymphocyte enumeration, including Tregs and class-switched/CD21low B-cell subsets. All patients should be included in standard population-based cancer screening programs. In pediatric patients, surveillance should also consider EBV assessment, with ultrasound (± PET–CT) for suspicious lymphadenopathy and biopsy in cases of persistent lymphoproliferation.

During follow-up, gastrointestinal surveillance should include annual non-invasive testing for *H. pylori* (urea breath test or stool antigen), periodic reassessment of vitamin B12, iron profile, and GPC and IF autoantibodies, and endoscopic surveillance guided by histological findings (intervals ranging from 6 months to 5 years). Hematological surveillance should include physical examination every 6 months with targeted lymph node assessment, total-body CT in cases of ≥5–10% unexplained weight loss over 6 months, and bone marrow aspiration and biopsy in the presence of persistent (>6 months) cytopenias or monoclonal gammopathy. When initial bone marrow assessment is unremarkable in the setting of persistent cytopenias, peripheral causes - including immune-mediated destruction, hypersplenism, and nutritional deficiencies - should be systematically excluded before considering repeat evaluation. Immunological monitoring should include annual advanced immunophenotyping.

Overall, implementing these harmonized surveillance pathways across clinical settings, supported by multidisciplinary collaboration and future prospective research, is expected to reduce practice variability, enable earlier diagnosis, and improve long-term outcomes for patients with CVID.

However, these recommendations should be interpreted with caution given several limitations. The document is expert-derived, and the proposed pathways are not supported by the highest level of evidence, as they do not originate from randomized controlled trials and have not been prospectively validated; accordingly, the parameters discussed reflect expert opinion rather than patient-level data. In addition, the panel was convened within Spain and the recommendations are anchored to the Spanish healthcare context and to the national CVID epidemiology represented by the participating experts and previous published registries, which may limit generalizability to other countries, healthcare systems, and patient populations, particularly where cancer patterns differ and may justify different surveillance priorities (10, 11). Likewise, implementation may be constrained by unequal availability of resources and care pathways across centers, particularly for advanced immunophenotyping and genetic testing, protocolized endoscopy, or access to virological testing, which may affect reproducibility. Additionally, the Delphi methodology did not allow for formal subgroup analysis by specialty, precluding statistical comparison of agreement levels between pediatric and adult-care specialists. Finally, prospective studies are needed to implement these recommendations in real-world practice and to evaluate whether harmonized surveillance pathways translate into measurable improvements in earlier detection and long-term outcomes.

## 5 Conclusion

This Delphi consensus complements existing IEI management guidelines by providing the CVID-specific oncologic surveillance framework not previously addressed (14–16). It represents the first multidisciplinary recommendations for malignancy screening and surveillance in this population. The panel reached broad agreement that structured cancer surveillance should be embedded in routine CVID care, given that malignancy is a leading cause of mortality among non-infectious complications. By standardizing screening and follow-up, these recommendations aim to reduce practice variability, improve risk recognition, and enable earlier detection through consistent longitudinal monitoring. In pediatric patients, surveillance should remain age-adapted and individualized, as malignancy risk and disease course may differ from adults. Future efforts should focus on prospective validation, refinement of risk stratification, and optimization of surveillance pathways across settings and patient subgroups to advance precision-oriented care.

## Data Availability

The datasets presented in this article are not readily available due to confidentiality restrictions. Requests to access the datasets should be directed to Marta Dafne Cabañero Navalón, marta.dafne.cabanyero@gmail.com.
